# DAG Expression: High-Throughput Gene Expression Analysis of Real-Time PCR Data Using Standard Curves for Relative Quantification

**DOI:** 10.1371/journal.pone.0080385

**Published:** 2013-11-18

**Authors:** María Ballester, Rubén Cordón, Josep M. Folch

**Affiliations:** 1 Department of Animal Genetics, Centre de Recerca en Agrigenòmica (CRAG), Consorci CSIC-IRTA-UAB-UB, Bellaterra, Spain; 2 Departament de Ciència Animal i dels Aliments, Universitat Autònoma de Barcelona (UAB), Bellaterra, Spain; 3 Department of Computer Science, Centre de Recerca en Sanitat Animal (CReSA), UAB-IRTA, Bellaterra, Spain; Mount Sinai School of Medicine, United States of America

## Abstract

**Background:**

Real-time quantitative PCR (qPCR) is still the gold-standard technique for gene-expression quantification. Recent technological advances of this method allow for the high-throughput gene-expression analysis, without the limitations of sample space and reagent used. However, non-commercial and user-friendly software for the management and analysis of these data is not available.

**Results:**

The recently developed commercial microarrays allow for the drawing of standard curves of multiple assays using the same n-fold diluted samples. Data Analysis Gene (DAG) Expression software has been developed to perform high-throughput gene-expression data analysis using standard curves for relative quantification and one or multiple reference genes for sample normalization. We discuss the application of DAG Expression in the analysis of data from an experiment performed with Fluidigm technology, in which 48 genes and 115 samples were measured. Furthermore, the quality of our analysis was tested and compared with other available methods.

**Conclusions:**

DAG Expression is a freely available software that permits the automated analysis and visualization of high-throughput qPCR. A detailed manual and a demo-experiment are provided within the DAG Expression software at http://www.dagexpression.com/dage.zip.

## Introduction

Real-time quantitative PCR (qPCR) analysis is the most common method to analyze gene expression due to the excellent sensitivity and specificity of PCR. It generates high-quality data without the requirement of additional validation. In fact, this methodology is applied to validate data obtained by higher throughput technologies such as microarray or RNA-Seq experiments [1]. Due to the technological advances produced in recent years, this methodology can also be used to perform high-throughput gene-expression quantification [2], [3]. In parallel to these technological advances, several methodologies have been developed to calculate the relative fold-change expression taking into account the efficiency of PCR [4]–[8]. Among them, the comparative threshold cycle method [4], [8] is one of the most commonly used methods and has recently been implemented in R packages for users with experience in this statistical environment [9], [10]. However, this method requires the PCR efficiencies of target and control genes to be approximately equal, and close to 100%, requirements that are not always achieved with high-throughput gene-expression measurements with qPCR. Other methods using different mathematical models, in which target-specific amplification efficiencies are introduced into equations to calculate relative expression data normalized to one [5] or multiple [7] reference genes, have also been developed. The latter is now available through the commercial software qBase^Plus^ (Biogazelle) [7].

It is well established that the best method to calculate PCR efficiency is through the construction of standard curves [7]. One of the advantages of the commercial microarrays currently available, such as the OpenArray® (Applied Biosystems) or the microfluidic dynamic Arrays (Fluidigm), is the feasibility of constructing standard curves of multiple assays using the same n-fold, serial diluted samples. Hence, this allows for the use of relative standard quantification to compare the relative concentrations among multiple samples and the analysis of gene-expression profiling among multiple assays. In the linear regression analysis method, the relative concentrations of unknown samples are calculated accounting for unequal efficiencies of target and control genes. Here, we have developed user-friendly software for the automated analysis of high-throughput gene-expression data by drawing relative standard curves for relative quantification, allowing the use of one or multiple-reference genes for sample normalization. To the best of our knowledge, there is no available software to perform an interactive analysis with standard curves with multiple genes and samples.

## Methods

### Implementation

Data Analysis Gene (DAG) Expression has been developed in Visual Basic.Net and will run under the major Microsoft operating systems (Microsoft® Windows® 7 or XP). A detailed manual and a demo-experiment (example results data) consisting of a microfluidic dynamic array™ IFC (48.48) containing 48 assays (44 target and 4 reference genes) and 48 samples are available in the **Help** menu, which allow users to be familiar with the DAG Expression software.

### Installation

The program does not need installation, it is zipped into a file and the user can unzip it with a standard program to a folder or desktop. In most systems, Framework.Net is installed, however, if the program gives an error message, the user must download and install Framework.Net 4 or above (http://www.microsoft.com/en-us/download/details.aspx?id=17851).

### Software features

The diagrams for processing analysis are presented in Figure S1 and S2. At present, the DAG Expression workflow can be summarized as follows:

#### Data import

**Figure 1:**
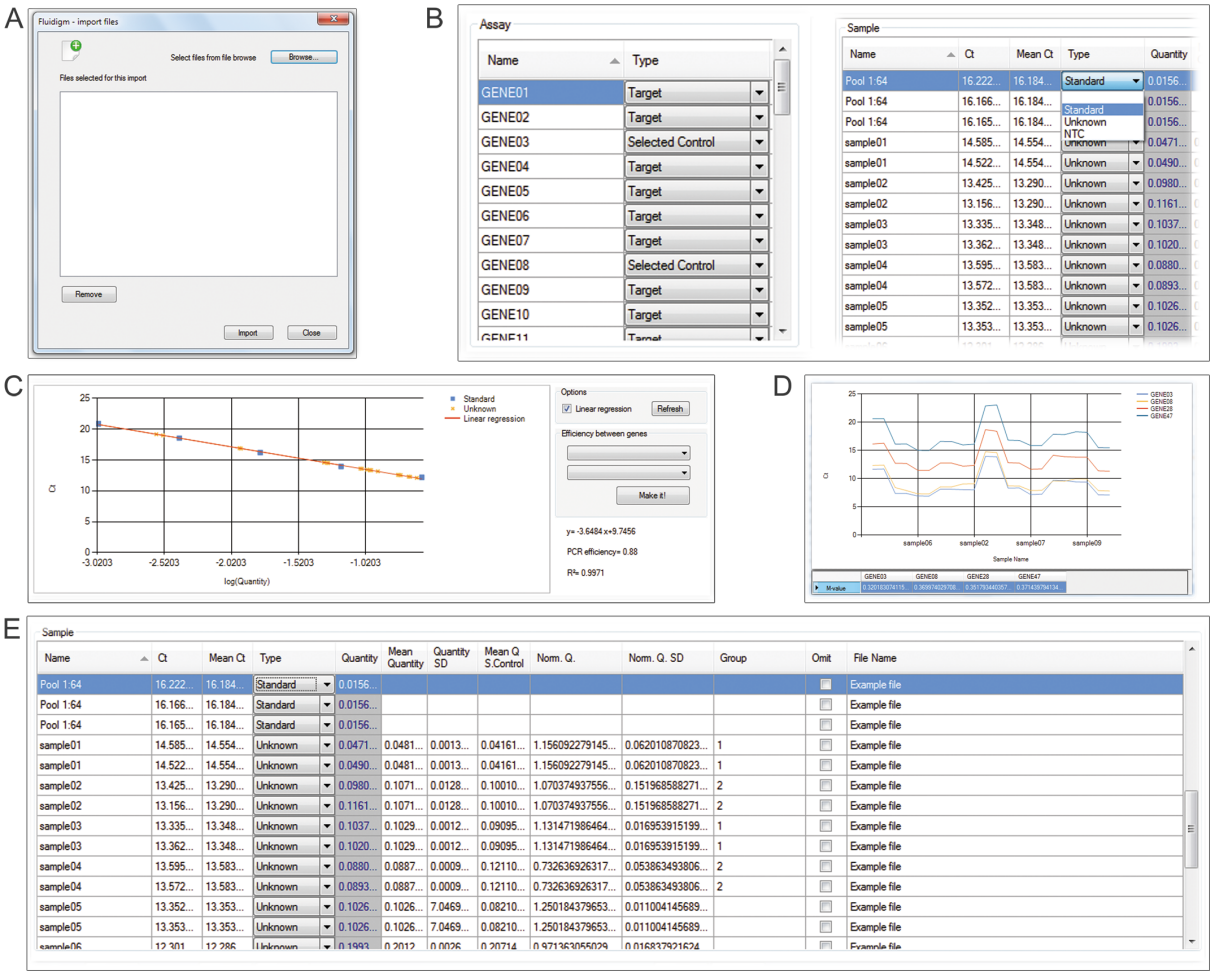
DAG Expression; (A) Import window; (B) Assay data table (left) and sample data table (right) work area; (C) Standard curve with a four-fold dilution series (1/4, 1/16, 1/64, 1/256, 1/1024) used to extrapolate the quantity values of Unknown samples; (D) Control-gene stability analysis. M-values for 4 selected control genes; (E) Results table with different parameters presented for each assay.

Output files (.csv or txt files) of different software (Fluidigm Real-Time PCR analysis software, SDS, etc.) or user-formatted input data containing the assay name (genes being measured), sample name and Ct (threshold cycle) values separated by a semicolon can be imported (Figure 1A). The program is able to import multiple files, allowing for the analysis of more than 96 genes and hundreds of samples; the name of each file can be visualized in the ‘File Name’ column of the sample data table (main work area).

Once imported, the DAG expression software converts these files into a standard internal format which contains information on the assay name, sample name (user can modify it), Ct value, and Mean Ct value (the arithmetic mean of the raw Ct values for the technical replicates of a given gene; samples with the same name are considered as being technical replicates by the program). Furthermore, when output files are imported to DAG expression, the software directly omits the data points that fail during the qPCR amplification. This allows the identification of these missing data points and allows users to further select data points and exclude them for subsequent analysis.

Two output files (fluidigm or generic csv) of the same demo-experiment have been included in the zip file to permit users to practice with the import action and to be familiar with the DAG Expression accepted formats.

#### Setting parameters

To start data analysis, different parameters such as the assay type (Selected Control or Target), the sample type (Standard, Unknown and Non-Template Control-NTC) and the quantity values in the Standard samples (serial dilutions) have to be set (Figure 1B).

#### Calculation of relative quantities

Once the raw qPCR data have been imported and the different parameters have been set, the software draws the standard curves for each assay interactively by plotting the Ct values (independent variable, Y) versus the log input amount (serial dilution values; dependent variable, X) of Standard samples (Figure 1C). Then, the software uses the standard curves to calculate the relative Quantity for Unknown samples (Step 1) applying the linear regression equation (y = mx+b) for the best fit line, where the slope m and intercept b are calculated as:
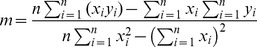


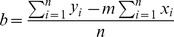
where y is Ct of the Standard sample, x is the quantity of the Standard sample in log, n =  number of technical replicates i.

*Step 1 (equations 1 and 2)*: Conversion of Ct values into relative quantities



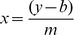
(Equation 1)




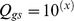
(Equation 2)where y is Ct of the Unknown sample, m is the slope of the standard curve, x is the quantity of the Unknown sample in log, b is the y-intercept of the standard curve line and 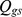 is the quantity of a Unknown sample (s) for a given assay (g): Selected Control or Target.

*Step 2 (equations 3 and 4)*: For all technical replicates, i, of an Unknown sample for a given assay, the software calculates the average quantity (Mean Quantity, 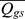) and the standard deviation (SD) of the average (Quantity SD, 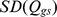).



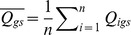
(Equation 3)where 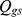  =  Quantity value of a Unknown sample for a given assay; n =  number of replicates i



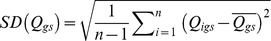
(Equation 4)where 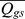  =  Mean Quantity value for all replicates of a Unknown sample for a given assay; n =  number of technical replicates i

#### Identification and selection of the most stable expressed reference genes

Single or multiple genes can be used as a normalizer. The multiple-gene normalization method is based on the principles and formulas described by [11] in which the lowest gene-stability measure (M) value indicates genes with the most stable expression (Figure 1D).

Once the user has selected the reference genes (Selected Control), the program calculates the arithmetic mean of the mean quantity values of the Selected Controls (Mean Q. S. Control) to produce a normalization factor (NF), step 3.

*Step 3 (equation 5)*: Calculation of the NF



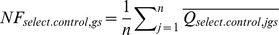
(Equation 5)where 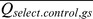  =  Mean Quantity value for all replicates of an Unknown sample for a given selected control; n =  number of selected controls j

#### Normalization of relative quantity values

Once the NF is obtained, the program calculates the normalized quantity (Norm. Q., 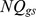) and the SD of the normalized quantity (Norm. Q. SD, 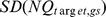) of all Unknown samples for each assay (Figure 1E), step 4.

*Step 4 (equation 6 and 7)*: Calculation of the normalized value and SD



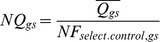
(Equation 6)

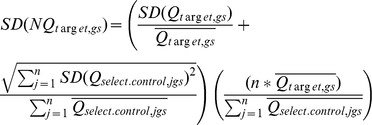
(Equation 7)where 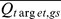  =  Mean Quantity value for all replicates of an Unknown sample for a given target assay; 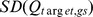  =  Standard deviation of the average quantity of a Unknown sample for a given target assay; 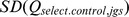  =  Standard deviation of the average quantity of an Unknown sample for a given selected control, n =  total number of selected controls.

#### Visualization of results

**Figure 2:**
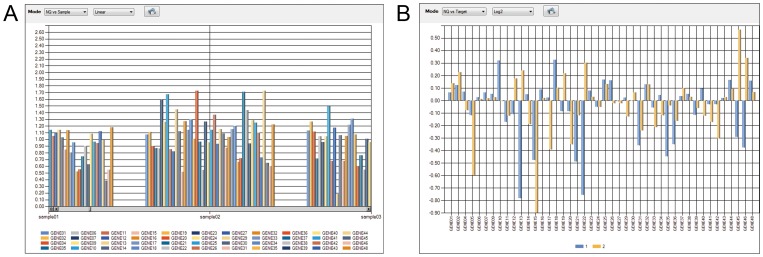
Bar chart example. (A) NQ plot displaying linear normalized quantity (NQ) vs sample. (B) NQ plot by group displaying logarithmic (Log_2_) normalized quantity (NQ) of each group (1 and 2) vs target.

DAG Expression normalized data can be visualized *via* bar plots (NQ Plot; Figure 2) displaying normalized data (Linear, Log_10_ or Log_2_) vs target or sample (Figure 2A). Groups of samples can be also created to visualize the data *via* bar plots; the software plots the arithmetic mean of the normalized data (Linear, Log_10_ or Log_2_) of each group vs target or group (Figure 2B).

Other utilities such as the “coefficient of variation inter-run” or the “efficiency between genes” are also available in the DAG expression software (See the user manual for more information).

## Results and Discussion

DAG Expression was created to analyze the expression of 48 genes (44 target genes and 4 reference genes) in 115 samples, using a 48.48 microfluidic dynamic array™ on the BioMarkTM system (Fluidigm) (Ballester et al., manuscript in preparation, 2013). However, the software can be used for the analysis of data obtained from different instruments (See data import in software features).

Data from our experiment were collected using the Fluidigm Real-Time PCR analysis software 3.0.2 (Fluidigm), and the output csv files were imported into DAG Expression, as is shown in Figure 1A. The demo experiment (Help menu: Example results data) shows the first experimental 48.48 dynamic array in which a seven-point four-fold dilution series (1/4, 1/16, 1/64, 1/256, 1/1024, 1/4096, 1/16389) per triplicate and 11 unknown samples per duplicate were run. Following “The MIQE Guidelines for Real-Time PCR Experiments” [12], standard curves covering at least 3 orders of magnitude were constructed for each gene (Figure 1C). PCR efficiencies were almost 90% for all of the assays except for Gene35, which was expressed at low levels and was discarded for further analysis. Reference genes Gene03 and Gene28 showed the lowest M value [11] (Figure 1D) and were selected as reference controls to perform subsequent analysis. To calculate the inter-run coefficient of variation, the same unknown sample was consecutively added in all the independent runs. We obtained a very small coefficient of variation (1.9%), indicating a good reproducibility of the microfluidic dynamic arrays (data not shown) [2]. Next, standard curves were used to extrapolate the quantities of the 115 unknown samples using linear regression analysis, and data were normalized using the previously calculated normalization factor (See the calculation step 3 in software features). At this point, the user can visualize the results using the Bar Plots tool to compare the relative expression levels among multiple samples or to view the expression profile of multiple genes. Furthermore, depending on the main aim of the study, data can be easily exported for further statistical and biological interpretation of gene expression data using other specialized programs.

**Figure 3:**
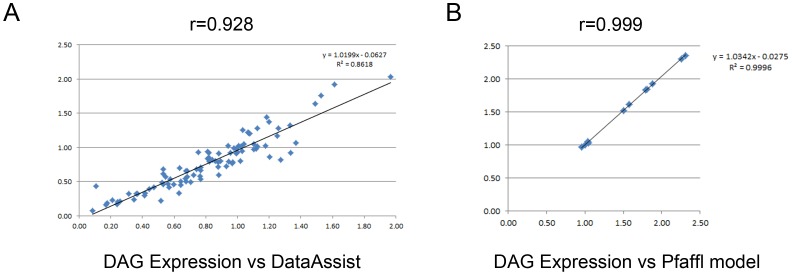
Comparisons of data. (A) Comparison of data obtained with the 2^−ΔΔCT^ method (DataAssist™) vs the relative standard quantification (DAG Expression). (B) Comparison of data obtained with the Pfaffl model vs the relative standard quantification (DAG Expression). The coefficient of correlation (r) is shown above each plot.

To assess the quality of the analysis performed by our software, a comparison with other methods [5], [8] was performed. First, we compared our results against results obtained using conventional qPCR on microliter volume samples and the comparative C_T_ (ΔΔC_T_) method [8]. In a previous study, the expression profile of the pig *ELOVL fatty acid elongase 6* (*ELOVL6*) gene was evaluated by qPCR using an ABI PRISM 7900HT Sequence Detection System (Applied Biosystems). Results were analyzed using the RQ manager v1.2.1 and the DataAssist™v3.0 software (Applied Biosystems) (for more details, see [13]). The same set of primers (*ELOVL6* and reference genes) was added into the 48.48 microfluidic dynamic array™ to be used as control. When the 2^−ΔΔCT^ values (DataAssist™) and the normalized quantity values (DAG Expression) of the same 94 samples corrected by the same calibrator sample were compared, a high correlation coefficient (r = 0.928) was obtained (Figure 3A). The subtle differences observed between the two methods are most probably due to variations in the qPCR amplification efficiencies obtained between experiments (conventional qPCR vs microfluidic array), although slightly differences between measurement platforms cannot be discarded [2]. In the former experiment, qPCR of *ELOVL6* and endogenous genes were optimized to obtain PCR efficiencies close to 2 and equal between target and endogenous genes. This optimization is time-consuming and expensive when profiling lots of genes using high-throughput technologies such as 48.48 microfluidic dynamic arrays and, for that reason, the method used by DAG expression, based on standard curves and linear regression analysis, corrects for differences in PCR efficiencies between target and endogenous genes without the need for further qPCR optimization. On the other hand, our demo-results were compared with the results obtained using the mathematical model developed by [5]. For this comparison, we used the target gene Gene17, the reference gene Gene03 and the control sample 11, obtaining also a high correlation coefficient (r = 0.999) (Figure 3B). In this case, both methods adjust for differences in PCR efficiency between target and internal control using different equations. Therefore, our software represents a reliable and accurate tool to perform relative quantification of high-throughput gene-expression data.

## Conclusions

In conclusion, recent advances in large-scale RT-qPCR platforms have allowed for the generation of a great amount of gene-expression data. Several methods to determine the relative gene-expression levels have been developed over the years. DAG Expression allows for the management and analysis of high-throughput gene-expression data-sets obtained by RT-qPCR using standard curves for relative quantification and one or multiple genes for sample normalization.

## Supporting Information

Figure S1 **DAG expression flow chart.** Workflow diagram for the general processing analysis of DAG expression.
(TIFF)

Figure S2 **Control gene stability flow chart.** Workflow diagram for the ‘find control gene stability’ tool.
(TIFF)
