# Supplementary figures and images for: DAG Expression: High-Throughput Gene Expression Analysis of Real-Time PCR Data Using Standard Curves for Relative Quantification
Source: PLoS One. 2013 Nov 18;8(11):e80385. doi: 10.1371/journal.pone.0080385 (PMC3832397; doi:10.1371/journal.pone.0080385)

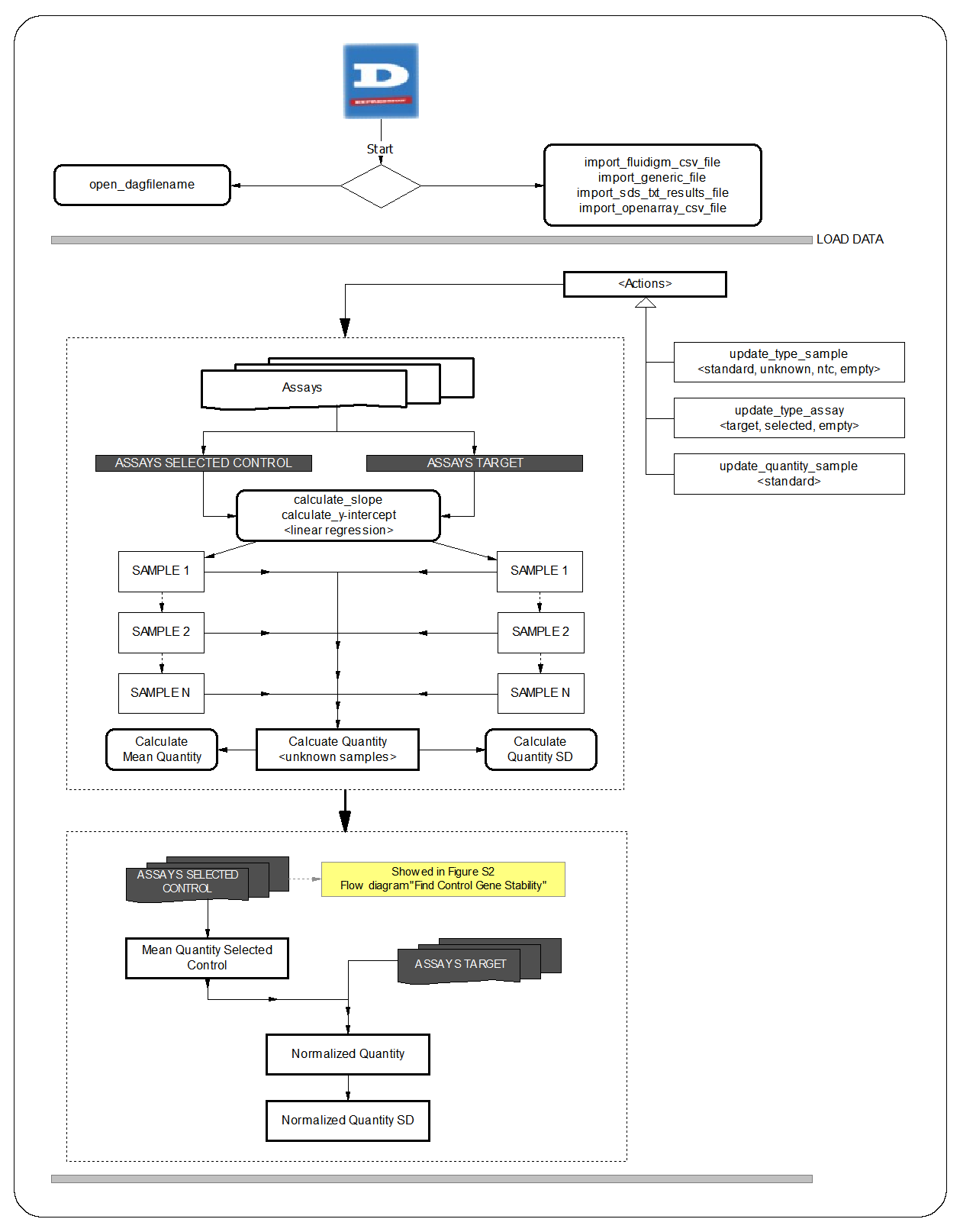

Supplement: Figure S1 — DAG expression flow chart. Workflow diagram for the general processing analysis of DAG expression. (TIFF) [file pone.0080385.s001.tiff]

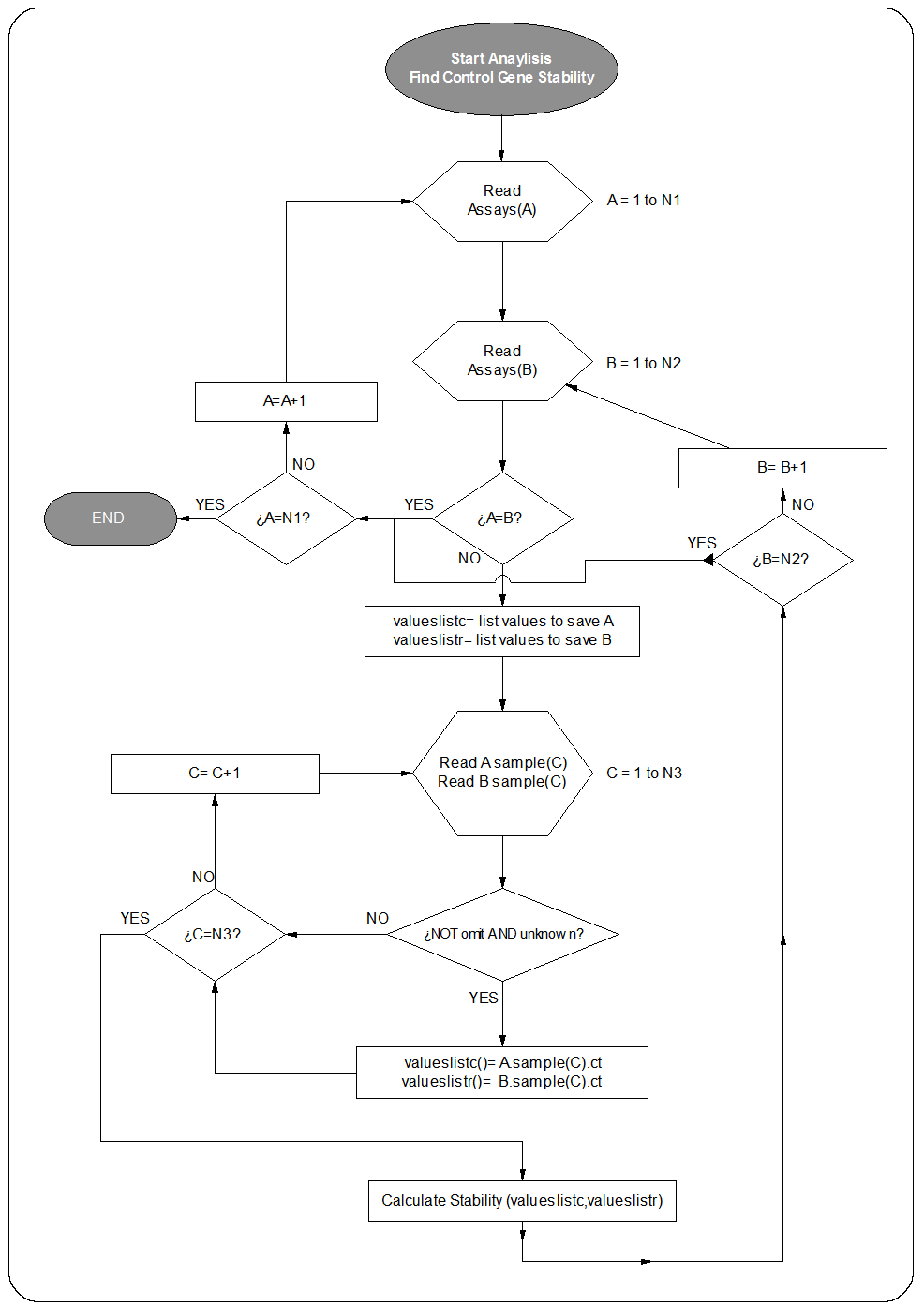

Supplement: Figure S2 — Control gene stability flow chart. Workflow diagram for the ‘find control gene stability’ tool. (TIFF) [file pone.0080385.s002.tiff]
